# Comparative Dosimetry of Handheld Versus Wall-Mounted Intraoral X-ray Systems: A Phantom-Based Study

**DOI:** 10.7759/cureus.100091

**Published:** 2025-12-25

**Authors:** Vignesh Guptha Raju, Rajsandeep Singh, Ameer Akhil Ahmed Shaik, Sai Priyanka Gaddipati, Venkat Hemant Akurati, Niti Dharmendra Shah, Rahul VC Tiwari, Seema Gupta

**Affiliations:** 1 Department of Pedodontics and Preventive Dentistry, Karpaga Vinayaga Institute of Dental Sciences, Chennai, IND; 2 Department of Dentistry, Punjab Civil Secretariat, Chandigarh, IND; 3 Department of Oral Medicine and Radiology, Narayana Dental College and Hospital, Nellore, IND; 4 Department of Dentistry, P.M. Nadagouda Memorial Dental College and Hospital, Bagalkot, IND; 5 Department of General Dentistry, School of Dentistry, University of Louisville, Louisville, USA; 6 Department of Global Management, Thunderbird School of Global Management, Phoenix, USA; 7 Department of Oral and Maxillofacial Surgery, RKDF Dental College and Research Center, Bhopal, IND; 8 Department of Orthodontics, Kothiwal Dental College and Research Centre, Moradabad, IND

**Keywords:** dosimetry, exposure, operator, radiation, safety

## Abstract

Introduction: The rapid adoption of handheld dental X-ray units has revolutionized imaging in mobility-restricted patients; however, concerns may still persist regarding their radiation safety profile compared with traditional wall-mounted systems. The aim of the present study was to quantitatively compare operator- and patient-equivalent radiation doses, ambient scatter, and diagnostic image quality between a handheld (MaxRay Cocoon, Dexcowin Co. Ltd., Korea) and a wall-mounted (Optima DC, Alerio Technologies Pvt. Ltd., India) intraoral X-ray unit during standardized mandibular molar radiography.

Materials and methods: This prospective experimental study was conducted using a human cadaver mandible as a standardized phantom. A total of 384 exposures (198 per device) were performed with identical exposure time (0.18 seconds), rectangular collimation, and a size-2 complementary metal-oxide-semiconductor (CMOS) digital sensor using the paralleling technique. Operator doses were recorded using real-time electronic personal dosimeters (RaySafe i3, RaySafe, Sweden) at the chest and thyroid levels and thermoluminescent dosimeters (TLD-100) placed under the lead apron at the chest, forehead, and fingers of the dominant hand. The patient-equivalent effective dose was calculated from dose-area product (DAP) measurements using conversion coefficients recommended by the International Commission on Radiological Protection. Image quality was evaluated objectively through signal-to-noise ratio (SNR) and contrast-to-noise ratio (CNR) measurements and subjectively by three blinded experienced oral radiologists using a five-point Likert scale. Data were analyzed using independent sample t-tests.

Results: The handheld unit produced significantly higher operator doses: finger dose 0.45 ± 0.12 µSv vs. 0.08 ± 0.03 µSv (p < 0.001), chest (outside apron) 0.18 ± 0.05 µSv vs. 0.05 ± 0.02 µSv (p = 0.001), and thyroid 0.11 ± 0.03 µSv vs. 0.04 ± 0.01 µSv (p = 0.003). The effective dose to the mandible phantom was 2.65 ± 0.25 µSv (handheld) vs. 1.82 ± 0.18 µSv (wall-mounted) (p < 0.001). The ambient scatter at 1 m was three times higher for the handheld device (p < 0.001). No significant differences were found in SNR (p = 0.125), CNR (p = 0.08), or subjective diagnostic quality scores (p = 0.65).

Conclusions: Despite comparable diagnostic image quality, the handheld dental X-ray unit delivered substantially higher radiation doses to both the operator and the patient-equivalent phantom than the wall-mounted system. Wall-mounted units should remain the standard where patient mobility permits, reserving handheld systems for clinically justified scenarios.

## Introduction

Dental radiography is a fundamental diagnostic tool in modern dentistry and is essential for the accurate diagnosis, treatment planning, and monitoring of oral and maxillofacial conditions [1]. Advances in intraoral imaging technology have expanded the range of available X-ray systems, with wall-mounted units historically serving as the standard owing to their reliability, consistent output, and ability to allow operators to maintain a safe distance or stand behind protective barriers during exposure [2,3].

However, the fixed installation of wall-mounted devices limits their applicability in clinical scenarios involving patients with restricted mobility, such as those in intensive care units, nursing homes, operating theaters, or receiving domiciliary care. In such settings, transporting patients to a dental operatory for radiography is often impractical or contraindicated, potentially delaying or preventing necessary endodontic or restorative treatment.

To address these challenges, portable and handheld dental X-ray devices have been developed and are increasingly adopted. These units offer significant advantages in terms of mobility, compact size, and the ability to acquire diagnostic images at the bedside or chairside without moving the patient [4]. When used correctly with appropriate shielding and collimation, handheld devices are designed to comply with radiation safety standards while maintaining image quality comparable to that of conventional wall-mounted systems [5,6].

A key concern surrounding handheld X-ray units is the operator’s unavoidable proximity to the radiation source during exposure, in contrast to wall-mounted systems, where the operator can retreat behind a lead barrier or to a distance of at least 2 m. This difference raises questions regarding whether operator and patient radiation doses are truly equivalent between the two types of devices, even when identical exposure parameters (kV, mA, and time) are applied [7]. Although modern handheld units incorporate internal shielding, rectangular collimation, and scatter shields, real-world clinical measurements are needed to confirm that occupational and patient doses remain within safe limits and are consistent with the As Low As Reasonably Achievable (ALARA) principle [8].

Previous studies of radiation leakage and scattered radiation from handheld dental X-ray devices have yielded conflicting results. Some investigators have reported significantly higher annual operator exposure with certain handheld models compared to wall-mounted units [9], while another study reported the average operator whole-body dose for 915 intraoral radiographs to be 0.047 mSv and concluded that, with proper technique and shielding, operator doses are negligible [10]. These discrepancies may arise from differences in the device design, shielding efficacy, operator positioning, exposure settings, and measurement methodology.

The present study aimed to quantitatively compare the radiation doses received by the operator during routine intraoral radiography of the human cadaver mandibles using a conventional wall-mounted dental X-ray unit and a handheld device. This investigation aimed to determine whether the use of handheld devices results in a higher operator dose than a wall-mounted unit, thereby contributing critical safety data to inform clinical practice, training protocols, and regulatory recommendations in contemporary dentistry.

## Materials and methods

This prospective experimental study was conducted in the Department of Oral Surgery in collaboration with the Department of Oral Medicine and Radiology, RKDF Dental College and Research Center, Bhopal, Madhya Pradesh, India, from February to May 2022, after obtaining approval from the institutional ethical committee (IEC No.: RKDF/DC/PG/2022/199) and adherence to the Declaration of Helsinki (2013). To eliminate the anatomical variability inherent in live subjects, all exposures were performed using a human cadaver mandible. This standardized phantom model provided a reproducible anatomy for accurate dose and image quality comparisons.

Two intraoral X-ray systems were evaluated: a wall-mounted unit (Optima DC, Alerio Technologies Pvt. Ltd., India; 65 kV, 7 mA, 200 kHz DC output) and a portable handheld device (MaxRay Cocoon, Dexcowin Co. Ltd., Korea; 70 kV, 1.7 mA, constant potential DC, equipped with a 0.4-mm rectangular collimator and integrated backscatter shield). A standardized exposure time of 0.18 seconds was used for both systems based on preliminary density-matching tests. A size-2 complementary metal-oxide-semiconductor (CMOS) digital sensor (Schick 33, Dentsply Sirona, USA) and the paralleling technique using a positioning system ensured a consistent geometry (Rinn XCP-DS, Dentsply Sirona Inc., North Carolina, USA).

During the handheld operation, the operator held the device at full arm length (~ 45-50 cm), positioned at 1350-1500 relative to the primary beam, while wearing a 0.5-mm lead (Pb)-equivalent apron and thyroid shield. For the wall-mounted unit, exposures were activated from behind a fixed 2-mm Pb-equivalent barrier. A total of 384 radiographs (198 from each source) were acquired based on the minimum sample size determined using G*Power software (version 3.1.9.2; Heinrich Heine University, Düsseldorf, Germany) for 80% power and 5% alpha error from a previous study by Doyle and Finney [11], who studied the entrance surface dose (ESD) of the wall mount and a digital X-ray machine.

The operator’s dose was measured using dual dosimetry. Real-time scatter exposure was recorded using electronic pocket dosimeters (RaySafe i3, RaySafe, Sweden) worn on the chest (outside the apron) and thyroid. Thermoluminescent dosimeters (TLD-100, Harshaw, Thermo Fisher Scientific, USA) were simultaneously placed under the apron at the chest, forehead, and dominant hand fingers to assess the dose to the protected and unprotected body regions. Environmental scatter exposure was monitored using fixed dosimeters placed 1 m distance from the source during imaging with each device.

In addition to operator exposure, the radiation dose to the human cadaver mandible was measured to represent the patient-equivalent effective dose. A calibrated dose-area product (DAP) meter (VacuDAP, VacuTec GmbH, Dresden, Germany) was attached to the X-ray tube head during phantom imaging in both systems to directly measure the ESD and DAP values. The effective dose (microsievert as µSv) for mandibular molar exposure was subsequently calculated using the DAP-to-effective dose conversion coefficients recommended by the International Commission on Radiological Protection (ICRP Publication 103) [12,13].

Image quality was evaluated both objectively and subjectively. For objective assessment, an aluminum step wedge was positioned over the phantom to quantify signal-to-noise ratio (SNR) and contrast-to-noise ratio (CNR) from predefined regions of interest using ImageJ software. Subjective evaluation consisted of a blinded observer study in which three experienced oral radiologists independently reviewed randomized images, including phantom images with simulated pathologies to mimic clinical diagnostic conditions. A standardized five-point Likert scale (1 = non-diagnostic, 5 = excellent) was used to assess overall diagnostic acceptability, contrast, sharpness, and noise [14]. The Likert scale is a freely usable public-domain psychometric tool for ordinal rating and requires no permission or licensing for research or clinical application.

All quantitative data were recorded in Microsoft Excel (Microsoft Corporation, Redmond, Washington, United States) and analyzed using the IBM SPSS Statistics for Windows, Version 23 (released 2015; IBM Corp., Armonk, New York, United States). Normality was evaluated using the Shapiro-Wilk test. The radiation dose and image quality outcomes were compared between the two systems using the independent samples t-test, with statistical significance defined as p < 0.05. Results are presented as the mean ± standard deviation.

## Results

Comparative analysis revealed significant disparities in radiation dose with comparable image quality between the two systems. The handheld unit demonstrated a substantially higher operator dose, with finger exposure (0.45 µSv) being over five times greater than the wall-mounted unit (0.08 µSv, p < 0.001). Similarly, operator exposure at the chest (outside the apron) and thyroid was significantly elevated using the handheld device (p = 0.001 and p = 0.003, respectively). The phantom mandible effective dose was also significantly greater for the handheld unit (2.65 µSv vs. 1.82 µSv, p < 0.001). Conversely, no statistically significant differences were found in any of the image quality metrics, including SNR (p = 0.125), CNR (p = 0.08), and subjective scores (p = 0.65). The ambient scattered radiation at 1 m was three times higher for the handheld device (p < 0.001). While the handheld unit offers portability, it delivers a significantly higher radiation dose to both the patient and operator without compensating for the improvement in diagnostic image quality. A wall-mounted unit is a more dose-efficient choice for fixed operating settings.

The results demonstrated a clear divergence between radiation safety and diagnostic efficacy. Operator dosimetry revealed significantly elevated exposure from the handheld unit at unshielded anatomical sites, including the chest (outside the apron), thyroid, and most notably, the fingers (p < 0.001), which was directly attributable to the operator's proximity to the scatter source. Conversely, doses under protective apparel and at the forehead were comparable, confirming the efficacy of personal shielding, regardless of the technique used. Furthermore, the handheld system delivered a significantly higher patient effective dose (2.65 µSv vs. 1.82 µSv, p < 0.001) and generated a threefold increase in ambient scatter radiation at 1 m (p < 0.001), indicating a less contained radiation field. Crucially, this increased dosimetric burden was not accompanied by any enhancement in image quality, as the objective (SNR, CNR) and subjective diagnostic parameters showed no statistically significant difference (p > 0.05). Collectively, these findings indicate that the wall-mounted unit offers superior dose efficiency for both patients and operators, achieving equivalent diagnostic image quality at a substantially lower radiation cost (Table 1).

**Table 1:** Comparison of operator radiation exposure, phantom effective dose, image quality metrics, and ambient scatter between a wall-mounted and a handheld intraoral X-ray system. Data are presented as mean ± standard deviation (SD) with 95% confidence intervals (CI), µSv: microsievert. Radiation dose outcomes represent operator exposure and phantom effective dose per single radiographic exposure. The chest (outside apron), thyroid, forehead, and fingers reflect unshielded or partially shielded anatomical locations. The chest (under the apron) reflects the protected dose beneath personal radiation shielding. A standardized five-point Likert scale (1 = non-diagnostic, 5 = excellent) was followed for subjective quality scoring [14]. *p < 0.05 was considered statistically significant using an independent t-test to compare groups.

Outcome measure	Wall-mounted unit (Optima DC) (n % = 192, 50%)		Handheld unit (MaxRay Cocoon) (n % = 192, 50%)		t-stats	p-value
	Mean ± SD	CI at 95% (lower limit, upper limit)	Mean ± SD	CI at 95% (lower limit, upper limit)		
Operator dose (µSv per exposure)						
Chest (Outside apron)	0.05 ± 0.02	0.04, 0.06	0.18 ± 0.05	0.17, 0.19	34.13	0.001*
Chest (Under apron)	0.01 ± 0.005	0.00, 0.02	0.01 ± 0.006	0.00, 0.02	0.02	0.45
Thyroid	0.04 ± 0.01	0.03, 0.05	0.11 ± 0.03	0.10, 0.12	31.3	0.003*
Forehead	0.02 ± 0.008	0.01, 0.03	0.03 ± 0.01	0.02, 0.04	1.75	0.32
Fingers	0.08 ± 0.03	0.07, 0.09	0.45 ± 0.12	0.43, 0.47	42.3	<0.001*
Phantom effective dose (µSv per exposure)						
Effective dose	1.82 ± 0.18	1.79, 1.85	2.65 ± 0.25	2.61, 2.69	38.1	<0.001*
Image quality						
Signal-to-Noise Ratio (SNR)	12.5 ± 1.2	12.3, 12.7	11.8 ± 1.5	11.6, 12.0	5.15	0.125
Contrast-to-Noise Ratio (CNR)	8.4 ± 0.9	8.2, 8.6	7.9 ± 1.1	7.7, 8.1	4.97	0.08
Subjective Quality Score (1-5)	4.3 ± 0.5	4.2, 4.4	4.2 ± 0.6	4.1, 4.3	1.81	0.65
Ambient scatter radiation (µSv per exposure)						
At 1 Meter	0.03 ± 0.01	0.02, 0.04	0.09 ± 0.02	0.08, 0.10	37.94	<0.001*

## Discussion

The present study provided robust experimental evidence that, under controlled and standardized conditions, the use of a handheld dental X-ray device resulted in significantly higher radiation doses to both the operator and the patient-equivalent phantom compared with a conventional wall-mounted unit, despite producing diagnostically equivalent images. These findings challenge the widespread assumption that modern handheld units with integrated shielding and rectangular collimation are dosimetrically comparable to wall-mounted systems when identical or clinically adjusted exposure parameters are used.

The most striking difference was observed in the operator finger dose, which was 5.6 times higher with the handheld device. This is because the operator’s hand is in close proximity to the exit port of the tube head and within the zone of maximum backscattered radiation during exposure. Even with a scatter shield, a fraction of the primary beam undergoes Compton scattering from the patient/phantom and returns to the operator’s hand when holding the device [15]. Similar elevated hand doses using handheld units have been reported by several authors. Danforth et al. [10] reported the highest operator dose for reproductive organs (0.0095 µSv), followed by the thyroid (0.0033 µSv) and fingers (0.00125 µSv), and Leadbeatter and Diffey [16] recorded right finger doses of 0.69 µSv and left finger doses of 0.78 µSv using the Rextar X (Posdion Co., Ltd., South Korea) camera-style hand-held dental X-ray unit. Makdissi et al. [17] recorded the highest radiation dose to the palm of the left hand (0.0310 mGy). The difference in radiation doses might be due to differences in methodology. In contrast, wall-mounted units entirely eliminate hand exposure because the operator activates the exposure remotely.

Operator chest (outside apron) and thyroid doses were also significantly higher in the handheld unit (approximately 2-3-fold), consistent with reports by Makdissi et al. [17], who attributed this to the absence of a fixed protective barrier and the operator standing within the scattered radiation field. Although the handheld unit produced significantly higher per-exposure doses, the absolute values remained low. Even with 4,000 annual exposures and no protective equipment, the extrapolated whole-body effective dose would be only ~1.0-1.2 mSv (<6% of the current 20 mSv/year occupational limit) and the extremity dose ~1.8 mSv (<0.4% of the 500 mSv/year limit). Compared with the older maximum permissible dose (MPD) of 50 mSv/year [12], this represents <2.5% of the limit. Thus, handheld devices pose no realistic risk of exceeding regulatory limits with proper use; however, the markedly higher dose compared with wall-mounted units represents an unnecessary deviation from the ALARA principle in fixed clinical settings.

Perhaps the 46% higher effective dose delivered to the mandibular phantom by the handheld unit is more concerning. This cannot be explained by the minor difference in tube potential (70 kV vs. 65 kV) alone, as a higher kV typically reduces the patient dose for a given image density when using digital sensors. The primary culprit appears to be the shorter source-to-skin distance inherent to handheld operation and the 0.4-mm focal spot collimator, producing a less uniform beam profile at a close range. Our DAP measurements confirmed a higher ESD with the handheld device, despite identical exposure times. In contrast to our findings, a previous study reported that the doses for handheld systems were significantly lower than those for wall-mounted systems. The average monthly dose for the handheld systems was 0.28 μSv vs. 7.86 μSv (deep dose equivalent) for the wall-mounted systems [5].

Importantly, no significant differences were found in the objective or subjective image quality parameters. This aligns with multiple recent investigations demonstrating the diagnostic equivalence between handheld and wall-mounted systems when collimation and digital sensors are used [4,18]. The ambient scatter at 1 m was three times higher with the handheld unit, reflecting the poorer containment of the radiation field. This has implications for nearby staff and accompanying persons in wards or nursing homes where shielding is often minimal. Smith et al. [9] reported the ranges for estimated annual air kerma across the handheld devices were 0.14-0.77 mGy for the median, 0.41-1.01 mGy for the mean, and 1.32-2.55 mGy for the maximum.

Clinical implications and limitations

Handheld dental X-ray units remain invaluable in situations in which patient transport is impossible (ICU, operating theater, domiciliary visits, forensic odontology, and humanitarian missions). However, in fixed dental operatories, the routine use of handheld devices cannot be justified on radiation safety grounds because wall-mounted units deliver equivalent diagnostic information at substantially lower doses to both patients and operators. Operators using handheld units must strictly adhere to the recommended techniques: maintain maximum distance and backward angulation (135^0^-180^0^), use the provided scatter shield, wear lead gloves when feasible, and consider ring dosimeters for extremity monitoring. Regulatory bodies and training programs should emphasize these differences rather than treat all intraoral units as equivalent.

Limitations of the study include the use of a cadaver mandible rather than live patients, potentially underestimating soft-tissue scattering; a single handheld model was tested, and results may not generalize to newer devices with improved shielding; exposure time was standardized rather than individually optimized for each image (though preliminary density matching was performed); and long-term cumulative dose projections assume consistent technique without additional protective measures. Future studies should incorporate multiple handheld brands, live-patient dosimetry, and real-world clinical workflows to further refine the safety guidelines.

## Conclusions

This study demonstrated that under standardized conditions for mandibular molar radiography, the handheld dental X-ray unit delivers significantly higher radiation doses to both the operator and the patient-equivalent phantom than a conventional wall-mounted unit, without any improvement in diagnostic image quality. Although handheld devices remain safe and indispensable for patients with limited mobility, their routine use in fixed dental operatories cannot be justified on radiation protection grounds. Wall-mounted units should remain standard in permanent clinical settings to ensure optimal adherence to the ALARA principle.
